# A microscope stage controlled by a BBC Model B microcomputer

**DOI:** 10.1155/S1463924685000335

**Published:** 1985

**Authors:** M. Nieman, J. Christie, W. Richardson, T. Wheldon

**Affiliations:** West of Scotland Health Boards Department of Clinical Physics & Bio-Engineering 11 West Graham Street Glasgow G4 9LF UK


**Journal of Automatic Chemistry**/*Journal of Clinical Laboratory Automation*, **Vol. 7, No. 3 (Jul–Sep 1985), pp. 155–157**

### A microscope stage controlled by a BBC Model B microcomputer

**M. Nieman, J. Christie, W. Richardson and T. Wheldon**

*West of Scotland Health Boards, Department of Clinical Physics & Bio-Engineering, 11 West Graham Street, Glasgow G4 9LF, UK*

### Introduction

A microscope fitted with a TV image analysis system (Microscope type Biostar: Reichert-Jung UK, 820 Yeovil Road, Slough, Berkshire SL1 4JB, UK; Image analysis type 40–10: Micromeasurements, Saffron Walden, Essex CB11 2AQ, UK) was being used by the West of Scotland Health Boards to investigate the growth pattern of cancer cells grown in the form of multicellular tumour spheroids. These are aggregates of cells, comprising up to a million cells depending on spheroid size, which are currently being explored as realistic models of small tumours (micrometastases) in the human patient. Spheroid response to radiation or chemotherapy is presently a subject of intensive investigation [1], and is studied by observing the growth of spheroids placed in individual small wells after treatment.

Spheroids were grown in 24- or 96-compartment multi-well plates and were examined periodically under the microscope. The spheroids can range in size from 200 to 1200 μm and a magnification of 40 times was used. Each well was examined in turn. The stage was moved until the spheroid was in the centre of the field of view. The image analysis system then measured the area of the spheroid.

The microscope users were sometimes unsure of which well they were examining. They also felt it would be better to have some remote handling method for the microscope because this would reduce the chance of microbiological contamination of the wells – this is a serious problem when working with human tumour spheroids. This procedure would also minimize any (theoretical) hazard to the operator from the tumour cells. A computer-controlled system was built using a BBC model B microcomputer, which allowed the multi-well plate to be indexed from one well to the next with a clear indication of which well was being studied. Other facilities were built into the software to provide a convenient system for the microscope user.

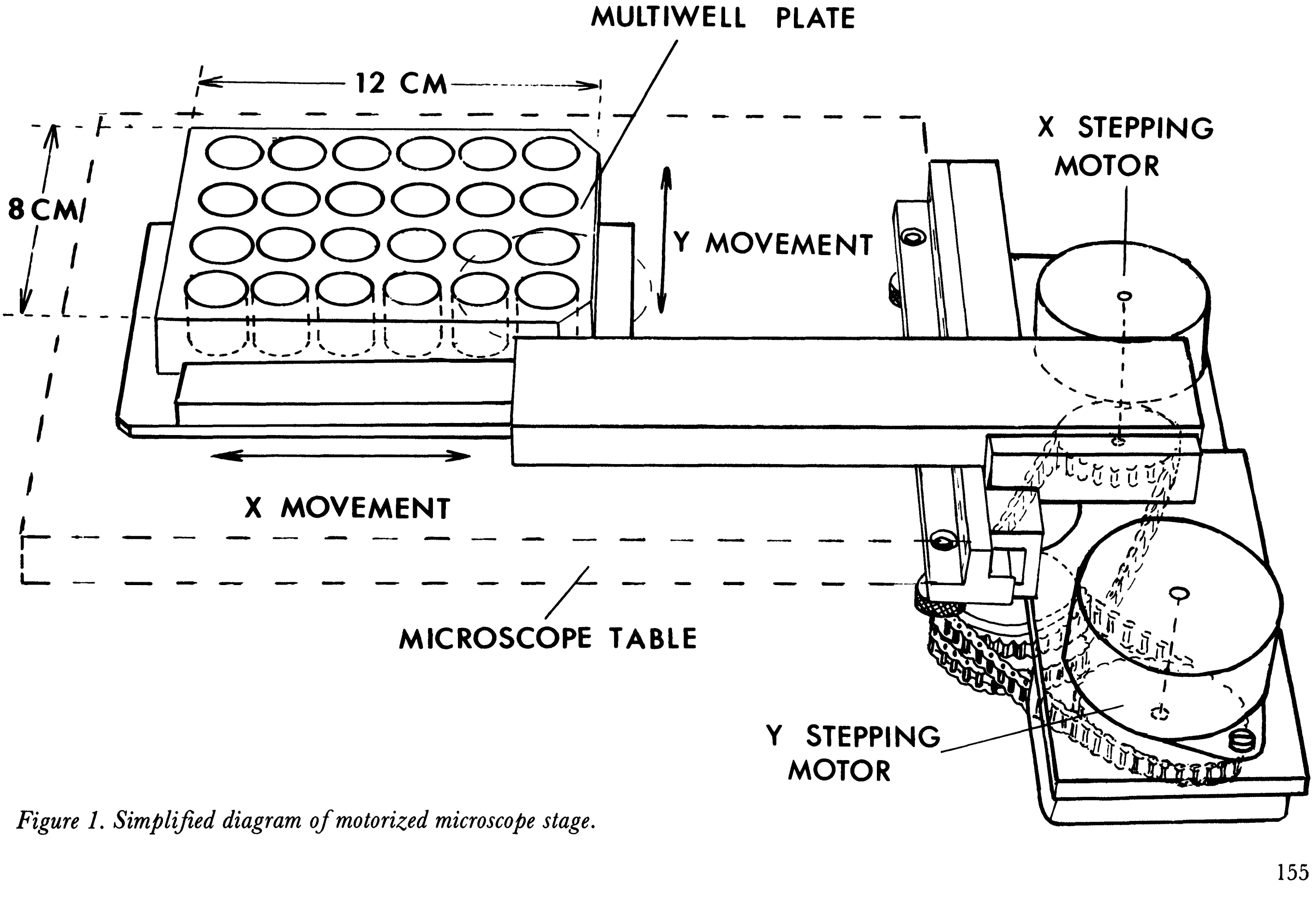

*Figure 1. Simplified diagram of motorized microscope stage.*

### Hardware

*Microscope stage and focus*

The manually operated stage supplied with the microscope was moved in $X$ and $Y$ directions by two co-axial knobs. These were replaced by adaptors fitted with plastic spur gears which were driven through lightweight plastic chains by stepping motors (figure 1).

The motors were unipolar, four-phase types, requiring 48 steps per revolution. They were fitted with 25:3 reduction gearboxes, so that resolution and output torque to drive the stage were both improved. The low-cost stepping motors used could not be driven above about 80 step/s without the output torque becoming too low to prevent steps being missed. The gearing gave adequate positional accuracy of 0.08 mm without significantly slowing the speed of travel of the stage.

Four microswitches, which operated at the limits of travel of the $x$ and $y$ movements, were fitted to avoid overtravel which could have damaged the mechanism.

To make the entire system capable of remote operation the focus on the microscope was also motorized using a small d.c. motor. No limit switches were used here, current limiting on the motor reducing output torque to a value which could do no damage. The focus can be adjusted by the microscope user operating a switch.

*Computer drive*

The stepping motors were driven through an interface connected to the user port of the BBC Model B. This computer has been described fully by Cowell [2].

For each motor only a direction signal and step pulse were required, the Signetics integrated circuit SAA1027 decoding these to provide the drive signals to the four coils of the stepping motor in the correct sequence. In the interface, the microswitch signals operated logic to prevent further movement when a limit of travel was reached. A signal was also sent to the user port to show that a microswitch had operated. To prevent overheating, another signal from the user port could de-energize the motor coils when the motors were not being used.

*Manual drive*

When the stage had been adapted for stepping motor drive it was no longer possible to move it manually. Another drive unit was provided for occasions when the BBC Model B was not available. This gave signals which mimic the output from the user port and so were accepted by the stepping motor drive interface. A joystick control enabled the stage to be moved in $x$ and $y$ directions. No read-out of position was provided, although the count up/count down pulse signals were available on a connector in the interface box and could be used for a display if this were ever necessary.

A 12V, 1A power supply was fitted into the manual drive unit. This is also used to power both the focus motor and the stepping motors even when they are being driven by the BBC micro as this keeps the computer cooler than when using the built-in disk supply. It also permits the use of disk drives with no independent power supply.

*System cost*

The cost of parts for the hardware required to motorize the focus and the existing stage was £760. This figure includes the cost of the BBC Model B, a cassette-recorder, and a monochrome monitor. The system is also available for other computing uses when not employed in driving the stage.

### Software

The use of a microcomputer to control the stage mechanism allows the controlling software to be developed quickly and makes the incorporation of modifications and additional features very simple. The program which controls the movement of the microscope stage was written entirely in BBC Basic and is about 10 K bytes long.

Because of the interface circuitry used, stepping the motors is achieved simply by writing one line of the user port high or low to select direction and then pulsing another line low to advance the motor by one step. A delay is built in to the stepping sequence to ensure that the maximum allowable step-rate is not exceeded.

Two more user port lines are configured as inputs to monitor the state of the microswitches. These are used to 'zero' the stage position before measurement on a new multiwell plate is commenced. This requires that the stage be driven towards the limits of travel in the $x$ and $y$ directions until the switch closures are detected. All subsequent stage movements are referenced to this position.

From the operator's point of view, the program is divided into three distinct modes of operation, each representing a different level of control (figure 2). Most of the program is concerned with handling these operating modes, the procedures for actually stepping the motors being quite straightforward. A commercially available joystick and switch handset was chosen as the means of selection and operation of the three modes so that the operator is freed as far as possible from the computer keyboard.

The first operating mode was written to meet the initial requirement of the control system, i.e. that it shold be possible to move from one well to another in such a way that the well currently in view is always properly identified. In this mode, the operator can move to the centre of the next or the previous well using two switches on the joystick handset or can specify any particular well on the computer keyboard and move to it directly. It is not possible to move around within a well in this mode. When the well centre has been reached, the operator has to move the stage until the the spheroid being cultured in that well is seen on the image analysis TV screen.

The second mode allows the operator to search systematically for the spheroid along a pre-defined search-path

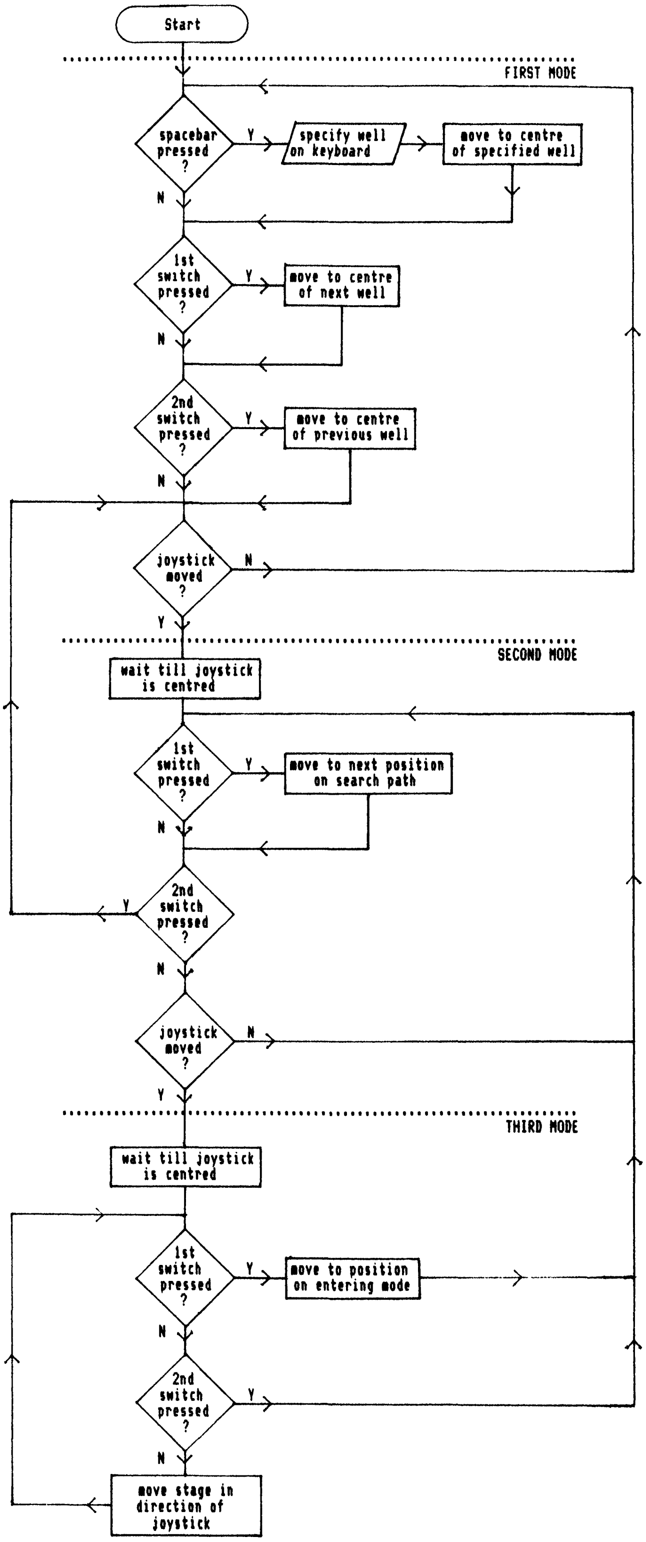

*Figure 2. Flow chart of microscope stage control program.*

within the currently selected well. To cover the whole area of the well as efficiently as possible, a 'square-spiral' search path was chosen, starting at the centre of the well since that is where the spheroid is usually found. The operator advances the field of view in discrete units along the path by pressing one of the handset switches, the units of movement being slightly less than the dimensions of the field of view to ensure minimum overlap between successive views.

When the spheroid has been found, the third operating mode can be used to centre the spheroid within the field of view ready for image analysis. In this mode the stage is moved directly in response to movement of the joystick by the operator. The various stages of image reversal present in the combined optical and TV system have been 'cancelled out': the joystick movement therefore corresponds in all directions to object movement within the field of view as seen on the TV display of the image analysis system.

In the second and third operating modes, movement is restricted to within the currently selected well to help ensure that the spheroids are properly identified with their corresponding wells.

At all times the identification number of the well being examined is displayed on the computer monitor.

### Discussion

The computer-controlled system has several advantages over manual operation. There is no confusion over which well is being examined. The operator only has to position the multiwell plate in the stage at the beginning of the read-out run and all other movements can be done remotely. This minimizes the chance of infection or contamination of the samples and the risk of spillage is reduced as all movements are smooth due to computer control. Systematic searching of the wells ensures that no spheroids are missed. Although the positional accuracy of the system is not as high as commercially available computer-controlled microscope systems, it met the requirements of the operators and was provided at relatively low cost.

### Acknowledgements

The technical support of J. Macleod is gratefully acknowledged.

### References

1. Twentyman, P. R., *British Journal of Cancer*, **45**, 565–570.
2. Cowell, T. K. *Journal of Medical Engineering & Technology*, **7**, 2, 79–82.

